# Additional evidence supports a timescale exceeding 100,000 years for the Guliya ice cap, Tibetan Plateau

**DOI:** 10.1126/sciadv.aeh2912

**Published:** 2026-09-30

**Authors:** Lonnie G. Thompson, Mary E. Davis, Ellen Mosley-Thompson, Juerg Beer, Tandong Yao, Christof Vockenhuber, Ninglian Wang, Ling Fang, Marcus Christl

**Affiliations:** ^1^ Byrd Polar and Climate Research Center, The Ohio State University, Columbus, OH 43210, USA.; ^2^ School of Earth Sciences, The Ohio State University, Columbus, OH 43210, USA.; ^3^ Department of Geography, The Ohio State University, Columbus, OH 43210, USA.; ^4^ Swiss Federal Institute of Aquatic Science and Technology, Dübendorf CH-8600, Switzerland.; ^5^ Key Laboratory of Tibetan Climate Changes and Land Surface Processes, Institute of Tibetan Plateau Research, Chinese Academy of Sciences, Beijing 100101, China.; ^6^ Laboratory of Ion Beam Physics, ETH Zurich, 8093 Zurich, Switzerland.; ^7^ College of Urban and Environmental Sciences, Northwest University, Chang’an District, Xi’an 710127, China.

## Abstract

The oxygen isotope (δ^18^O_ice_) record from a ∼310-meter core drilled on the Guliya ice cap (GIC) in 2015 (GP2015) demonstrates reproducibility with a ∼309-meter core drilled nearby in 1992. Construction of the timescale for a composite of the GIC cores used ^10^Be and ^36^Cl to locate the 41–thousand year Laschamp geomagnetic excursion and a match to a δ^18^O_atm_-dated core from the Guliya Summit. Comparison of the δ^18^O record from an Asian Monsoon speleothem with the GIC δ^18^O_ice_ supports a timescale back to 128 thousand years, the oldest known so far from Tibet. The δ^18^O_ice_ record shows orbital variations in phase with June insolation and polar ice core stable isotope records through the last glacial cycle, although the relationship is reversed since the deglaciation. Future investigation is required to resolve this observation. Radiocarbon-dated ice from the glacier margin supports the presence of glacial-stage ice in the GIC. The ^36^Cl/^10^Be ratios in GP2015 confirm that GIC ice is older than 100 thousand years.

## INTRODUCTION

Ice sheets and glaciers are unique natural archives of environmental changes with the oldest extending over 800 thousand years (kyr) back in time (*1*–*4*). They contain extensive climatic and environmental information, such as precipitation derived from the ice accumulation rate, temperature derived from the ratio of oxygen isotopes (δ^18^O_ice_), and atmospheric chemistry. Sporadic events such as volcanic eruptions, fires, and droughts may also be recorded. The value of the information gleaned from glaciers and ice sheets depends on their geographic location, the time intervals they represent, and the temporal resolution of those time intervals. However, the utility of this treasure trove of information depends on the accuracy of the ice core’s timescale, and dating these cores can be challenging. For older portions of cores, one of the most common dating methods uses the decay of radioactive isotopes such as ^14^C which depends on locating sufficient well-defined organic material in the ice, which is often sporadically available in small quantities and rare in polar ice cores. In addition, as ^14^C has a half-life of 5700 ± 30 years, its use is restricted to ages up to ∼55 ka. These constraints can result in timescales being constructed using a limited number of data points.

The glaciers and ice sheets on the Third Pole, composed of the Tibetan Plateau (TP) and its surrounding mountain ranges, provide a vital source of water for over a billion people from Central to South Asia (*5*). Since the middle of the 20th century, the cryosphere and hydrosphere of this vast region have become increasingly threatened by tropospheric warming. The glaciers on the western Kunlun Mountains in the northwestern TP contribute water to the rapidly developing Tarim Basin region. Until recently, the glaciers in this region have been expanding despite recent warming (*6*), in contrast to the shrinking cryosphere in the Himalayas. However, satellite imagery confirms that, since 2015, the glaciers in the northwestern TP have also been losing mass (*7*, *8*), likely in response to the recent, persistent increase in the length of the ablation season in this region (*9*). Therefore, the retrieval of ice cores from these threatened glaciers and ice sheets, along with the development and interpretation of their archived climate records, is a major objective. However, the critical first step in the interpretation of ice core data is the construction of an accurate timescale.

The Guliya ice cap (GIC) is the largest glacier in the western Kunlun Mountains and is located on the southern slope of the range (Fig. 1, A and B). It is a polar-type glacier which is frozen at the base (*10*, *11*). The GIC Summit (GS; 6710 masl) is located north of a large, flat ice plateau (6200 masl) that covers ∼132 km^2^ (*12*). The ice on the GS is much thinner than on the GIC Plateau (GP) (∼51 versus ∼370 m) (*13*). The western Kunlun Mountain region is cold and arid, and estimates of the amount of annual precipitation received by the GIC in recent decades vary between 106 and 428 mm water equivalent per year (mm w.e. a^−1^) (*9*, *11*, *13*). During a cooperative program in 1992, the Lanzhou Institute of Glaciology and Geocryology and the Byrd Polar and Climate Research Center (BPCRC) drilled a 308.7-m ice core to bedrock on the GP where the average accumulation rate is 0.28 ± 0.11 m w.e. a^−1^ (*13*). The results from the analysis of that core (GP1992) revealed a climate record older than 110 ka based on ^36^Cl (half-life 3.01 × 10^5^ years) concentrations and δ^18^O_ice_ matching to polar stable isotope and gas records (*14*) and raised the potential of a timescale that extends past ∼500 ka. In 2015, the BPCRC and the Institute of Tibetan Plateau Research (ITP) returned to the GIC and not only redrilled the GP to bedrock (GP2015, 309.7 m) but also drilled three cores to bedrock on the GS (50.7, 50.9, and 51.4 m) where the accumulation is 0.27 ± 0.11 m w.e. a^−1^ (Fig. 1C) (*13*). The timescale for one of the GS cores (GSC3) was developed using δ^18^O_atm_ and ^14^C ages of discrete plant fragments in the ice and was transferred to another summit core (GSC2) to create a summit ice core composite (GS2015) (*15*). The 15,000-year δ^18^O_ice_ record from this composite (fig. S1A) was matched to the GP2015 δ^18^O_ice_ record, and along with the ^36^Cl and ^10^Be (half-life 1.39 × 10^6^ years) data from the GP2015 core, a 41-kyr record was constructed for the top 187.4 m of GP2015 (fig. S1B). This timescale supports the original conclusion from the GP1992 timescale (*14*) that the GIC contains glacial-stage ice (*16*).

**Fig. 1.**
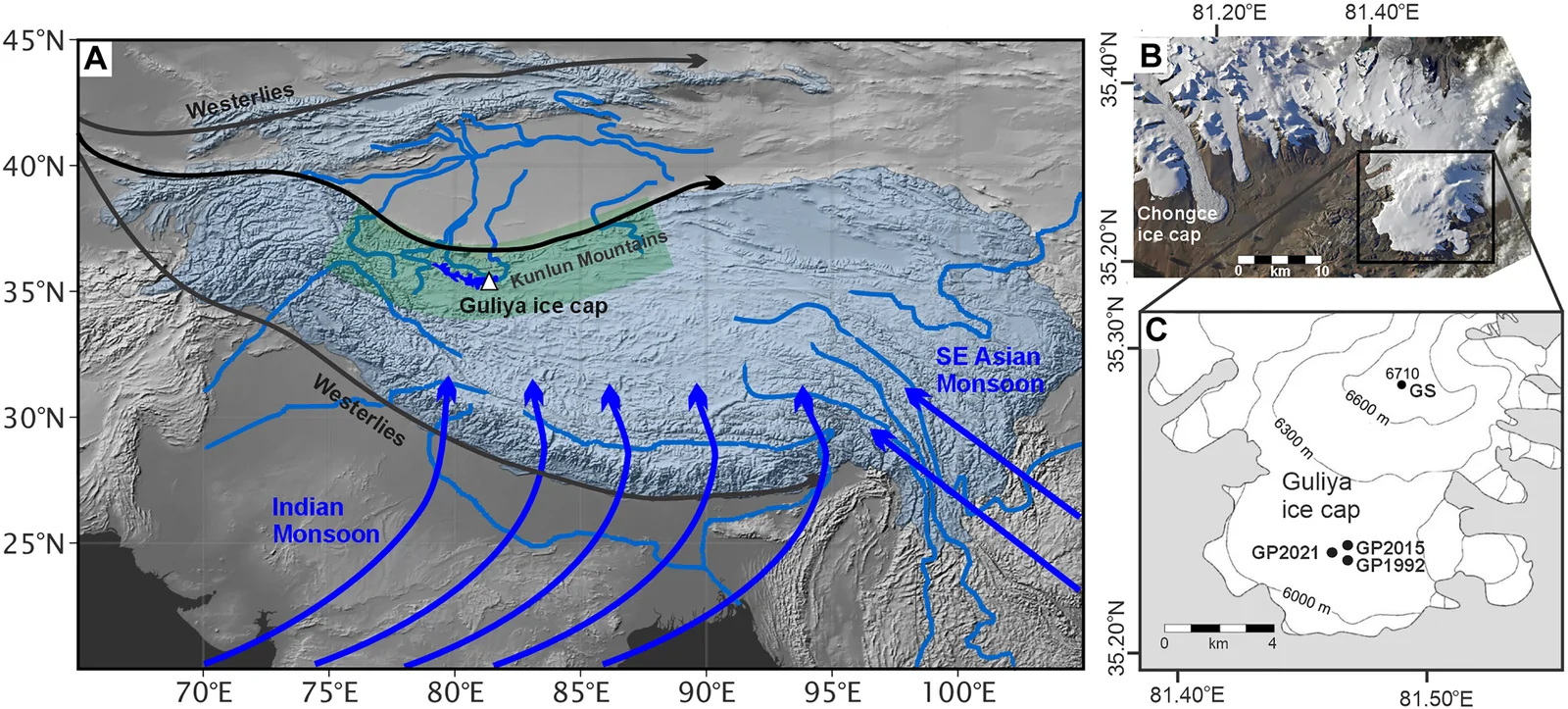
Geographic setting of the GIC. (**A**) Relief map of the TP (highlighted in light blue) showing major rivers and the movement of major air masses, along with the locations of the GIC (white filled triangle) in the western Kunlun ice sheet (highlighted in darker blue). The Kunlun Mountain range is highlighted in green. (**B**) Satellite image of the southeast sector of the western Kunlun ice sheet. The GIC is outlined by the black box; the Chongce ice cap to the west is marked. (**C**) A topographic map of the GIC shows the locations of the 1992, 2015, and 2021 drill sites on the Guliya Plateau and the drill site on the Guliya Summit. Reprinted with minor modifications from (*16*) with permission from Elsevier.

Over the past decade, several publications have challenged the age of the Guliya ice core record (*17*–*22*) and claimed that the GIC is no older than the Mid-Holocene. Ice cores collected from the Chongce ice cap near the GIC in the West Kunlun ice sheet (Fig. 1B) and an ice core drilled on the GIC in 2021 in close proximity to the 1992 and 2015 drill sites (Fig. 1C) show a wide range of Holocene ages (*19*–*22*). The Chongce ice cap, located just 30 km west of the GIC, was drilled to bedrock in 2012. Measurements of δ^18^O_atm_ yielded a maximum age of ∼9.8 ka at 215.61-m depth (*21*), and a spliced ∼7000-year δ^18^O_ice_ record was produced from two cores using radiocarbon dating of water insoluble organic carbon (^14^C-WIOC) (fig. S1C) (*19*). In 2021, the Guliya Plateau was drilled again, and a 175-m ice core was recovered from a site less than 1 km west of the 1992 and 2015 boreholes (Fig. 1C) (*22*). Although this new core was drilled very close to the earlier core sites, the ice at the 2021 site (∼230 m) is much thinner than the 1992 and 2015 sites (308.7 and 309.7 m, respectively) due to the bedrock topography, and the accumulation was determined to be 0.2 ± 0.05 m w.e. a^−1^. Thus, the length of that ice core is ∼75% of the total ice depth at the drill site. Using radiogenic isotopes (^210^Pb, ^39^Ar, and ^14^C-WIOC), the age of the core at 175 m was determined to be of Late Holocene age (2.7 ka) (fig. S1D), which is considerably younger than the ages of the GP1992 and GP2015 records at stratigraphically equivalent depths (*22*) and of the 15 ka GS2015 cores. In comparison, the record of δ^18^O_ice_ for GP1992 was constructed to 1.65 ka (fig. S1E) by counting visible annual dust layers to 109 m below which they were no longer distinguishable (*23*). As 1.65 ka is 199 m above the bedrock, the complete GP1992 record must be much longer (and hence much older) than that from GP2021.

Here, the chronology of GIC ice cores is extended through the last glacial cycle independently of the methodology used to establish the GP1992 timescale (*14*). In addition, ^14^C-WIOC ages of ice sampled from the margin of the GIC support the presence of ice older than the Holocene in this ice cap.

## RESULTS

### Extending the GP timescale beyond 41 ka

The most recent 41 kyr of the GP2015 δ^18^O_ice_ record (*16*) shows significant correlations with the absolutely ^230^Th-dated speleothem δ^18^O records of Indian and Southeast Asian Monsoon (SEAM) intensity (*24*, *25*). In particular, the SEAM speleothem δ^18^O composite record from caves in SE China (*25*) affords the opportunity to further extend the timescale of the GP ice cores. The GP2015 timescale has now been extended back to 128 ka (fig. S2) using (i) Analyseries software (*26*) to match the GP2015 δ^18^O_ice_ record before 41 ka to the SEAM speleothem δ^18^O record (*25*), (ii) the match between the δ^18^O_ice_ records from GP2015 and the δ^18^O_atm_-dated GS2015 record (*15*), and (iii) ^10^Be and ^36^Cl peaks at 187 m marking the location of the 41 ka Laschamp geomagnetic excursion (LGE) (*16*). Using the SEAM record to extend the Guliya timescale has two advantages: (i) The cave deposits were dated using ^230^Th which has a half-life of 75,690 ± 230 years (*27*), and (ii) the composite δ^18^O record of stalagmites from SE China is continuous, thus facilitating the match with the continuous GP2015 δ^18^O_ice_ record.

Like GP2015, GP1992 was analyzed at high resolution for δ^18^O_ice_ (*14*). The δ^18^O_ice_ records of the two cores show a very high degree of similarity (fig. S3A) and can be stratigraphically matched by depth (fig. S3B). Thus, the 128-kyr timescale for the GP2015 δ^18^O_ice_ data is easily transferable to the GP1992 δ^18^O_ice_ record (fig. S3C). The resulting timescales for both cores are very close to that for the original GP1992 which was developed by matching the δ^18^O_ice_ record to polar stable isotope and gas records back to 110 ka (*14*), with the largest difference noted between ∼1 and 22 ka (fig. S3C). Averaged intervals of δ^18^O_ice_ in both cores were combined to create a composite isotope record (GP1992/2015) (fig. S4). Both GP1992 and GP2015 were sampled throughout their entire lengths for ^36^Cl (46 samples from GP1992 and 34 samples from GP2015) (fig. S5, A and B). Each ^36^Cl sample from GP2015 was also measured for ^10^Be (fig. S5C). The depth match between the GP1992 and GP2015 δ^18^O_ice_ records also allows the ^36^Cl data throughout both cores to be combined in stratigraphic order, which essentially increases the sample resolution of the entire record (Fig. 2A). Using the timescale from the GP1992/2015 δ^18^O_ice_ record, the ^36^Cl data from both cores and the ^10^Be values from GP2015 can be placed in time back to 110 ka (Fig. 2B).

**Fig. 2.**
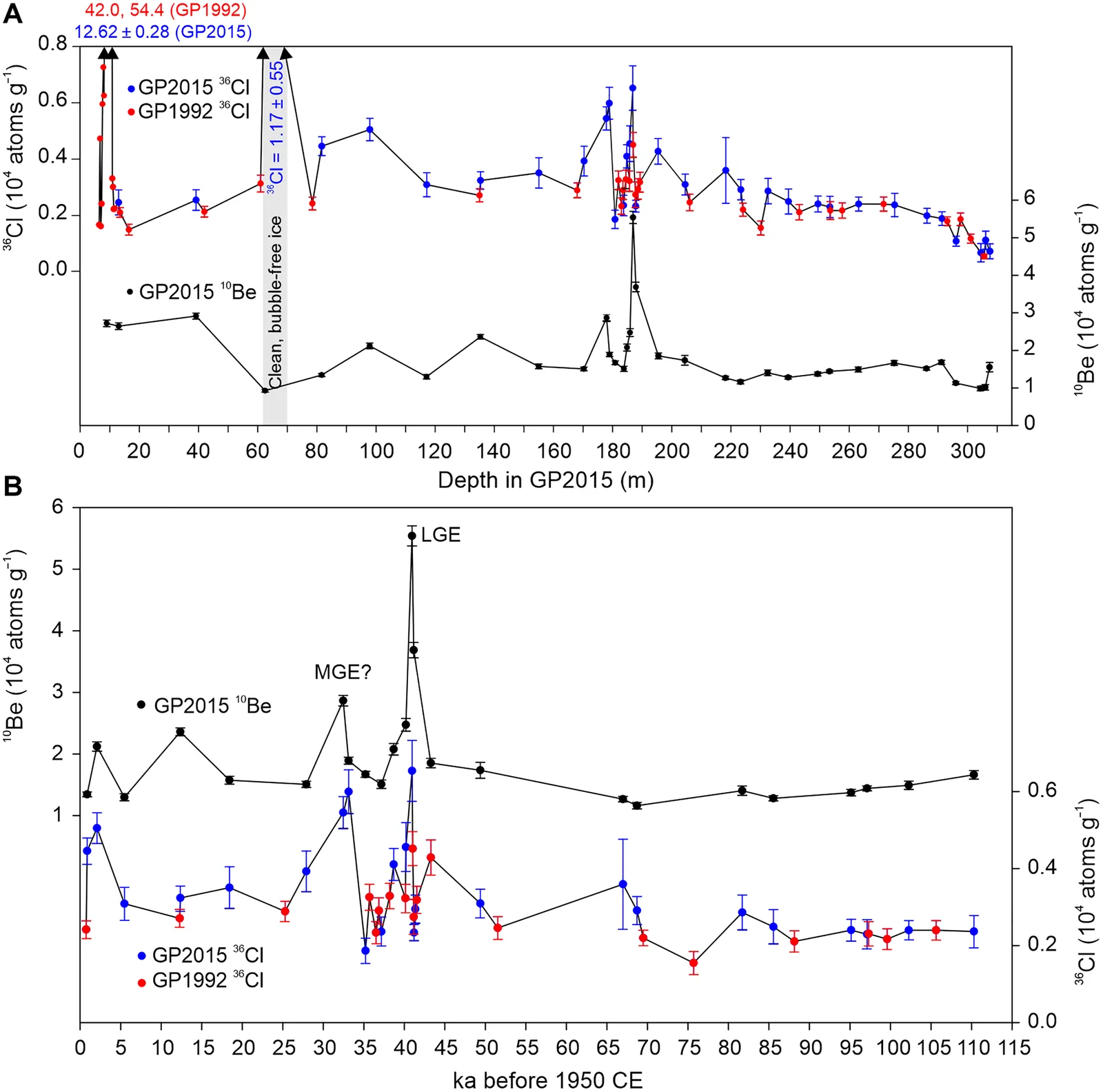
Cosmogenic isotopes in GP1992 and GP2015. (**A**) Integrated ^36^Cl values for GP1992 (red filled circles) and GP2015 (blue filled circles) and ^10^Be values for GP2015 (black filled circles), shown by depth. The elevated ^36^Cl values near the glacier surface reflect fallout from the 1952–1958 marine nuclear weapons testing in the South Pacific (*40*, *41*). (**B**) ^36^Cl and ^10^Be values shown in time over the last 110 kyr. The ^36^Cl and ^10^Be data from both GP1992 and GP2015 are measured values, i.e., they were not decay corrected. The Mono Lake Geomagnetic Excursion (MGE) and the LGE are noted. The large ^36^Cl concentrations shown at the top of the ^36^Cl depth plot in (A) are excluded.

Although not used in constructing the GP1992/2015 timescale, radiocarbon ages of ice collected at the margin of the GIC show that the ice cap is much older than the Holocene. During a 2015 field program conducted by the ITP, an ice block was collected from an excavated cavity ∼50 cm above the base of a vertical ice cliff on a lobe extending from the southeastern margin of the GIC (fig. S6), and two samples were prepared for ^14^C-WIOC dating following the protocols described in (*28*). The resulting calibrated age ranges of the two samples are 19,190 to 27,394 years before the present (yr B.P.) and 19,825 to 28,996 yr B.P., with mean calibrated ages of 24,071 ± 2321 yr B.P. and 24,833 ± 2577 yr B.P., respectively (fig. S7), placing the age of the margin ice one half meter above bedrock well into the last glacial stage.

## DISCUSSION

### Millennial to orbital variations in the GP1992/2015 δ^18^O_ice_ record

The GP1992/2015 composite that extends through the last glacial cycle captures the climatic features on millennial to orbital timescales that appear concomitantly in regional speleothem δ^18^O and polar stable isotope records (Fig. 3). Apart from Marine Isotope Stage (MIS) 1 (0 to ∼14.5 ka), the variations in summer insolation at 30°N (*29*) correspond directly with the GP1992/2015 δ^18^O_ice_ and deuterium excess (d-excess) records, showing high (low) insolation during high δ^18^O_ice_ and d-excess (low δ^18^O_ice_ and d-excess) stages (Fig. 3, A to C), while the speleothem records (Fig. 3, D and E) show an antiphase relationship with insolation and GP1992/2015 and polar δ^18^O_ice_ (Fig. 3, F and G). The discontinuous profile from the Ton Cave (*30*), which is located ∼1300 km northwest of the GIC in an arid region of Central Asia, contains large gaps in MIS2 and MIS4 (Fig. 3E) that occur during periods concurrent with low June insolation when reduced water seepage into the cave inhibited speleothem growth.

**Fig. 3.**
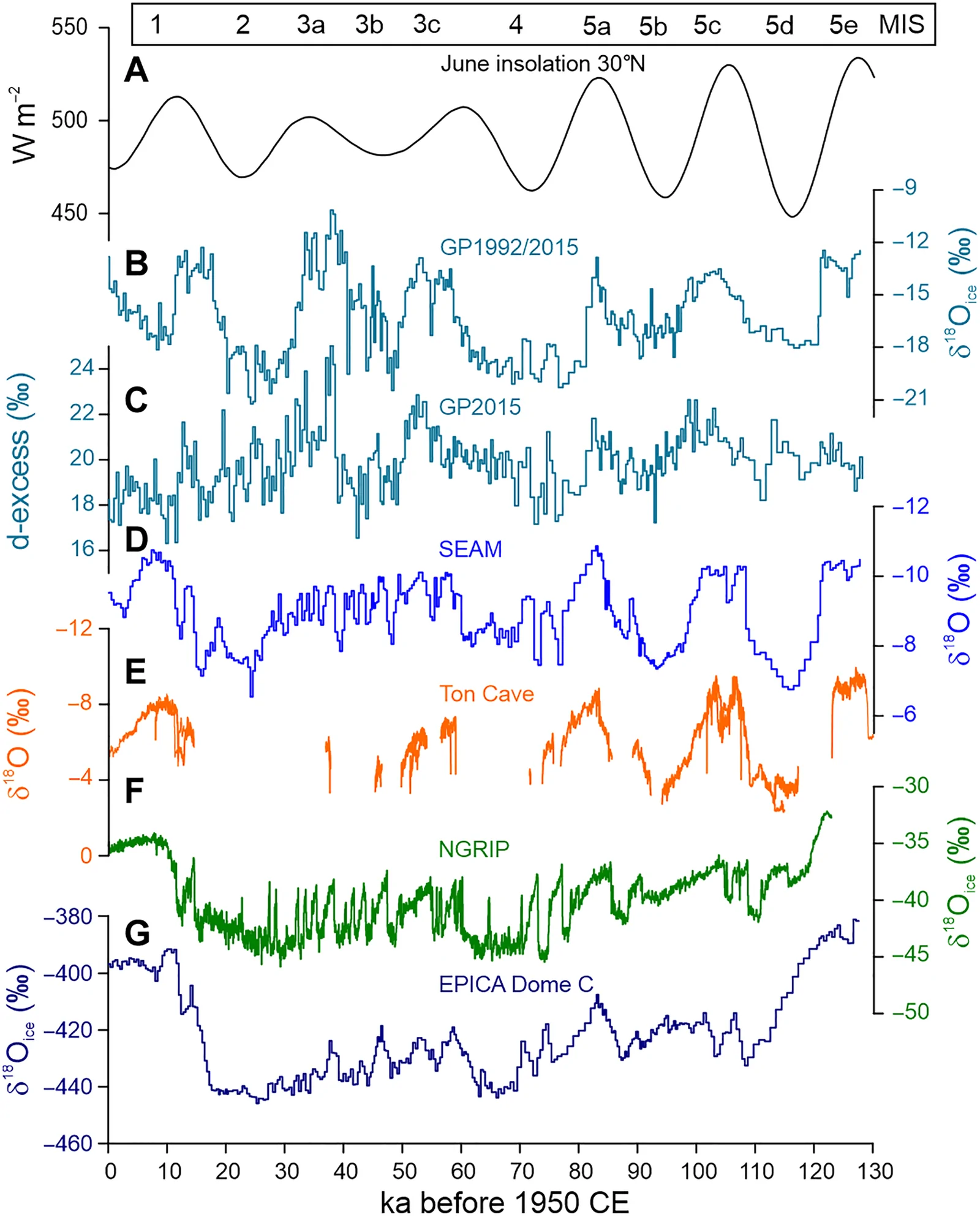
Asian and polar stable isotope records through the last glacial cycle. (**A**) June insolation at 30°N (*29*) from MISs 1 to 5e. (**B**) The δ^18^O_ice_ record from the GP1992/2015 composite is compared with (**C**) the d-excess record from GP2015; (**D**) the δ^18^O record from the SE Asian Monsoon speleothem composite (*25*); (**E**) the discontinuous δ^18^O record from Ton Cave, Central Asia (*30*); (**F**) δ^18^O_ice_ profile from North Grip (NGRIP), Greenland (*2*) and (**G**) the δD record from the EPICA Dome C ice core, Antarctica (*4*).

The antiphase relationship between GP1992/2015 δ^18^O_ice_ and the cave δ^18^O through the last glacial cycle (MIS2 to MIS5e) is expected if temperature is an important control on oxygen isotope fractionation in precipitation on the northern TP (*11*, *19*, *31*–*35*). Modeling of δ^18^O_ice_ shows that temperature is the primary control on isotopic fractionation over low and middle latitude glaciers whose surfaces, like Guliya’s, lie in the middle and upper troposphere (*36*). However, δ^18^O in cave deposits are more closely related to precipitation intensity, as is the case in speleothems in the Asian Monsoon region (*25*) and are subject to some alteration by carbonate precipitation from drip water (*37*).

The relationship between GP1992/2015 δ^18^O_ice_ and d-excess and the speleothem and polar ice core records observed from MIS2 to MIS5e reverses ∼18 to 19 ka (Fig. 3) (*16*). At the same time, the Guliya d-excess and δ^18^O_ice_ trends diverge (Fig. 3, B and C), i.e., as d-excess decreases toward the present the δ^18^O_ice_ switches from an antiphase to an in-phase relationship with the cave records. The reasons for this are unknown but can be speculated to result from changing boundary conditions in the North Atlantic and the Asian Monsoon region as the Northern Hemisphere climate emerged from the Last Glacial Maximum (LGM) ∼18 ka. Both GP1992/2015 δ^18^O_ice_ and summer insolation remained high during the deglaciation from the end of the LGM to the beginning of the Holocene (∼18 to 12 ka). However, as the Arctic ice sheets began to retreat and the westerlies and Asian Monsoon front shifted northward, the changing interaction between these dominant atmospheric circulation systems may have altered the moisture source and/or decreased the intensity of moisture recycling over the northern TP (*16*, *38*, *39*) as indicated by the divergence of the d-excess and δ^18^O_ice_ in GP2015 ∼18 to 19 ka (Fig. 3, B and C) that occurs simultaneously with the phase change between the ice core and speleothem oxygen isotopes (Fig. 3, B and D). Further research, including δ^18^O_ice_ modeling, is necessary to gain insight into stable isotopic fractionation and climate forcing in this region that lies under the influences of both the Asian Monsoon and the westerlies.

### Laschamp and Mono Lake geomagnetic excursions in GP1992/2015

Cosmogenic radionuclides such as ^10^Be and ^36^Cl share a common production signal, as their formation in Earth’s upper atmosphere is modulated by changes to the geomagnetic field, as well as by variations in the intensity of the solar wind. The only other source of ^36^Cl is thermonuclear testing at sea level, which occurred extensively in the South Pacific between 1952 and 1962 (*40*). Testing continued at a lower frequency until 1971, but fallout from the extensive 1950s/1960s tests continued into the early 1970s (*40*, *41*). This explains the high ^36^Cl concentration at ∼10.5 m in GP2015 which is unsupported by ^10^Be (Fig. 2A and fig. S5, B and C) and the exceptionally high ^36^Cl concentrations (up to 54 × 10^4^ atoms g^−1^) in a continuously sampled interval near the top of GP1992 (Fig. 2A and fig. S5A) (*42*).

Another unusually high concentration of ^36^Cl that occurs with a low ^10^Be concentration is observed in GP2015 at 62.5 m. This appears within an interval from 59 to 68.3 m in which glassy, bubble-free ice contains lower average dust concentrations and δ^18^O_ice_ compared to the ice above and below (fig. S8). It is unlikely that an almost 10-m-thick layer of meltwater formed either on the surface and later froze and was buried or that a 10-m section of ice melted and refroze at depth. However, as the GIC is located very close to active fault zones in a region with a history of intense seismic activity (*43*), this stratigraphic disruption may have resulted from the opening of a crevasse during an earthquake which allowed snow and/or summer meltwater near the surface that was contaminated with ^36^Cl from thermonuclear testing to be transported downward. This scenario is supported by the absence of ^10^Be, indicating that this ^36^Cl peak was not caused by a solar or geomagnetic event. The earthquake would have occurred at the time the bomb testing was underway (1950s to early 1960s) and might not have been detected or recorded because that region of the TP was very sparsely populated.

One of the arguments advanced against the GP1992 and GP2015 chronologies (*22*) involves the identification of the LGE dated at ∼41 ka which is marked by elevated ^36^Cl concentrations at 183 m in GP1992 and 187 m GP2015. However, unlike the ^36^Cl peaks in the shallower parts of the core discussed above, the ^36^Cl events at 179 and 187 m in GP2015 also show the increases in ^10^Be that would be expected from higher cosmic ray–induced production in the upper atmosphere. Thus, the ^36^Cl and ^10^Be spikes at 187 m in GP2015 support the original premise that the ^36^Cl spike at 183 m in GP1992 (fig. S5) corresponds to the LGE at ∼41 ka (Fig. 2B) (*44*–*46*). A comparison of the ^10^Be concentrations from the GP2015 and Greenland (GRIP) ice cores (fig. S9, A and B) (*46*) shows that the ∼41-ka event is the most prominent from 20 to 50 kyr, while the ^10^Be flux peak at the time is the highest in the last 60 kyr. In addition, the cosmogenic isotope data from Antarctica illustrate that the highest ^10^Be peak in the last 150 kyr occurs at ∼41 ka (*47*). The cosmogenic isotope peak at ∼179 m in GP2015 (Fig. 2A and fig. S5, B and C) is tentatively identified as the Mono Lake geomagnetic excursion (∼33 ka) (Fig. 2B), which has been observed in numerous records around the world (*45*), including those from ice cores (*48*).

The larger enhancement of ^10^Be over ^36^Cl at ∼185 m in the Guliya ice cores is characteristic of geomagnetic events rather than solar energetic particle events (SEPs) (*49*). SEPs are very short-term (hours to days) bursts of high-speed particles accelerated by solar flares that produce cosmogenic radionuclides in the atmosphere. Several intense events during the Holocene, such as a SEPs that occurred at 774 and 993 CE (*49*), have been observed in polar ice cores. Another indication that the cosmogenic peak in Guliya is from a geomagnetic rather than a solar event is the duration of the peak. As the LGE lasted ∼3 kyr (39.5 to 42.5 ka) and the cosmogenic signal in the Guliya record covers ∼10 m, a considerably shorter Guliya timescale extending only through the Late Holocene (*22*) would reduce the duration of the peak to ∼100 years which requires multiple SEPs to occur in one century. However, only a few large SEPs are known to have occurred over the last ∼12 kyr (*50*).

The δ^18^O_ice_ data around 41 ka may also give some additional support to the recording of the LGE in the GIC cores. Recent research into the linkages between Earth’s major geomagnetic excursions and climate indicates that such events may cause substantial changes in ozone concentrations and atmospheric circulation which trigger such global-scale climatic shifts. In particular, climate models of simulated surface temperature anomalies during the LGE (and concurrent grand solar minima) indicate that cooler climate associated with conditions that resemble the negative phases of the Arctic and North Atlantic Oscillations prevailed in much of the Northern Hemisphere, including Central Asia and the TP (*51*). This may explain the concurrence of large ^18^O depletions (cooler temperatures) in GP1992 and GP2015 with the cosmogenic isotope peaks at ∼41 ka (fig. S10).

### Dating GP1992/2015 beyond 128 ka

The distinct decrease in ^36^Cl concentrations below 290 m (fig. S5A) in GP1992 is used as evidence for very old ice (>500 ka), assuming a constant ^36^Cl production rate and no other influences on the ^36^Cl concentration excluding radioactive decay (*14*). The same ^36^Cl trend was reproduced in the GP2015 core (Fig. 2A and fig. S5B). However, a controversy regarding the postdepositional mobility of H^36^Cl surrounds the use of ^36^Cl to date ice cores at depth. The evidence for ^36^Cl loss (or no ^36^Cl loss) due to H^36^Cl outgassing or diffusion is based on the movement (or lack of movement) of the 1950s/1960s ^36^Cl peak resulting from nuclear weapons testing (*52*). For example, results from a ^36^Cl analysis of snow pits at Vostok Station, East Antarctica show that the concentration peaks over a decade later than expected (1958–1960 CE) based on the annual dating of the snow. Postdepositional processes may have led to H^36^Cl outgassing; thus, ^36^Cl may be unreliable for age determination in ice cores from low ice accumulation regions (*52*). Where ice accumulation is higher, such as West Antarctica, the ^36^Cl nuclear testing signal appears at 1958–1960 CE. This indicates that ^36^Cl loss is not significant, and therefore, the dating methodology using ^36^Cl (i.e., ^36^Cl/^10^Be) is sound (*53*). However, the Guliya ice cores present a different set of conditions from those in Antarctica. In the GP1992 core, which was sampled at high resolution in the upper 10 m for both ^36^Cl and beta emissions from the decay of nuclear explosion products ^90^Sr and ^137^Cs (*42*), the peak in the former occurs 60 cm below the 1963 peak in beta activity or ∼1960–1961 CE (fig. S11). If the criterion for lack of H^36^Cl mobility that is postulated for the Antarctic cores applies to the Guliya ice cores, then the use of ^36^Cl to determine the oldest part of the Guliya timescale is potentially sound although the ice accumulation at the GP drill site is low (∼28 cm/year) (*10*, *13*), although not as low as at Vostok Station (∼2 cm/year) (*54*).

Other than postdepositional processes, variations in production rate can also affect ^36^Cl concentrations in ice. However, when ^10^Be as well as ^36^Cl data are available, the ^36^Cl/^10^Be ratio compensates for the production rate changes (*55*). Figure 4 shows that the decrease of the ^36^Cl/^10^Be ratio in GP2015 starts at ∼220 m, 70 m above the depth where Cl^−^ concentrations decrease. Under the assumption that radioactive decay alone alters the ^10^Be and ^36^Cl concentrations, the ^36^Cl/^10^Be ratio can be used to calculate ages with an effective half-life of 384 kyr (*55*), as indicated by the right axis of Fig. 4, thus confirming the old ages close to the bottom of the GP1992 ice core (*14*). However, at the very bottom of the ice core, the concentrations of both ^10^Be and ^36^Cl have potential issues with respect to the mobility of ^10^Be and loss of ^36^Cl. If the ^36^Cl/^10^Be ratio is corrected for the Cl^−^ concentration, then the four lowest samples would result in unrealistically high ^36^Cl/^10^Be ratios (0.24 to 1.4) and would have to be interpreted as an overcorrection. Although age estimates based on ^36^Cl concentrations as well as the ^36^Cl/^10^Be ratio indicate that the ice could be significantly older than 100 ka, this observation is based on several assumptions (*55*), and thus establishing a timescale for the GIC ice cores that exceeds 128 ka remains a challenge.

**Fig. 4.**
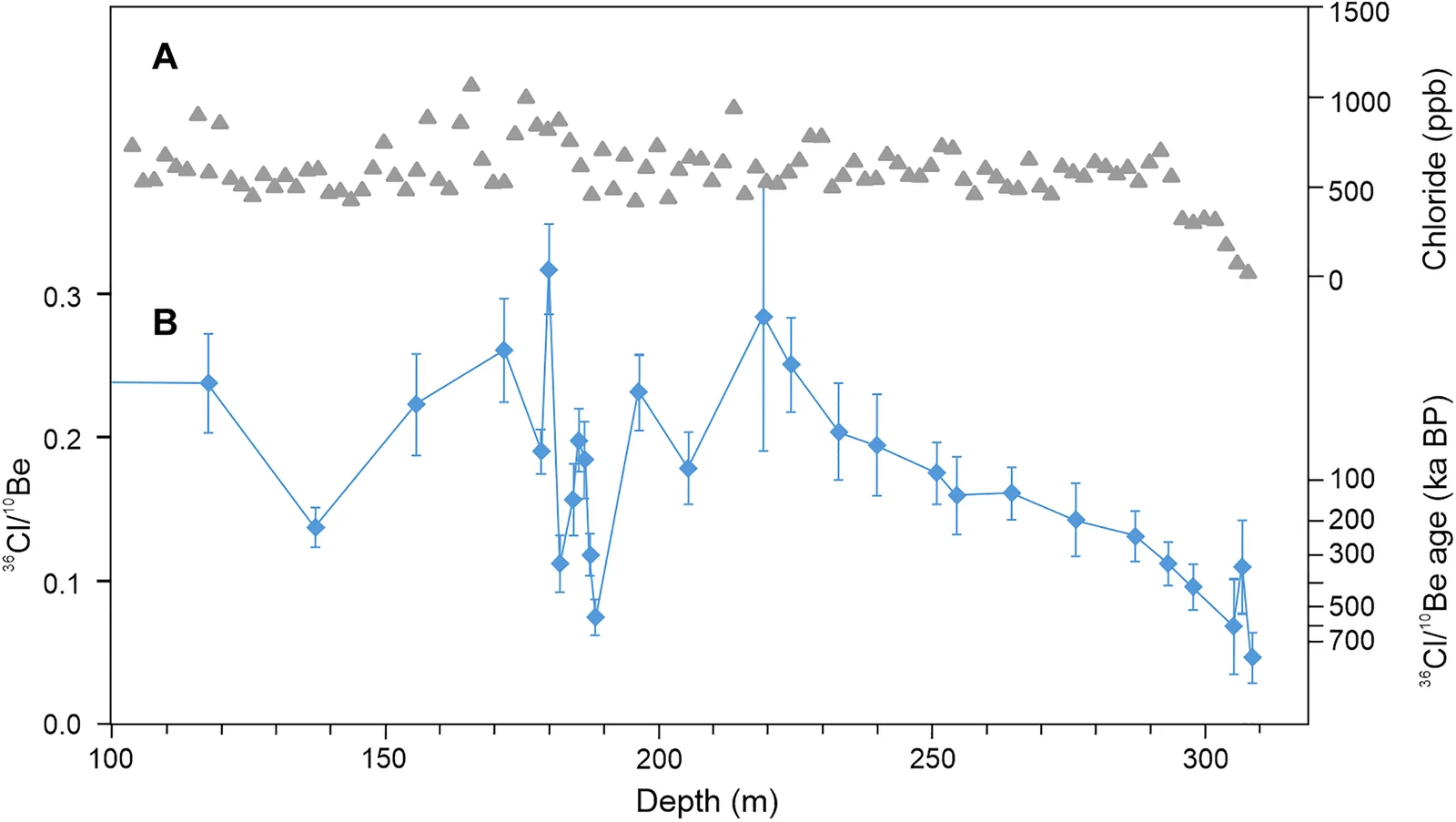
Chloride concentrations and ^36^Cl/^10^Be in GP2015. Concentrations of (**A**) Cl^−^ (gray triangles, 2-meter avgs) and (**B**) ^36^Cl/^10^Be ratio (blue diamonds) below 100-m depth from GP2015. The ^36^Cl/^10^Be age scale (right axis) is based on the initial ^36^Cl/^10^Be ratio of 0.204 (average of layers between 80 and 218 m) and radioactive decay with an effective half-life of 384 kyr (*55*). ppb, parts per billion.

The very large discrepancies between the radiocarbon-dated Chongce and GP2021 records (fig. S1, C and D) and between the GP2021 (fig. S1D) and cosmogenic isotope-dated GP2015 record (fig. S1B) indicate that additional methods of dating of TP ice cores should be explored. One such method that is gaining popularity is ^81^Kr dating of ice. With a half-life of 229 ± 11 kyr, ^81^Kr is a noble gas nuclide which is produced in the stratosphere by cosmic rays and has a residence time spanning hundreds of thousands of years. Therefore, it is an appropriate isotope for dating ice from ∼30,000 to 1 million years. Recent advances in ^81^Kr dating require only 1 kg of ice rather than the higher amounts (∼10 kg) previously needed to make a measurement (*56*). This would increase the feasibility of using ^81^Kr to date the bottom of the Guliya Plateau in the event that additional cores are drilled to bedrock. At the same time, the measurement precision would be improved, and age uncertainties reduced.

## MATERIALS AND METHODS

A cooperative field program on the GIC between the BPCRC and the Lanzhou Institute of Glaciology and Geocryology in the summer of 1992 resulted in the recovery of a 308.7-m ice core to bedrock. The core was transported by truck to Ürümqi where it was split equally along its length between the two institutes. The half-core allocated to BPCRC was returned frozen to the cold storage facility at The Ohio State University where it was maintained at −35°C. The core was cut into 12,628 samples for δ^18^O_ice_ and other parameters (e.g., dust concentrations and major anions). The δ^18^O_ice_ samples were measured on a Finnegan Mat Delta-E mass spectrometer with a precision of ±0.2 per mil (‰). The original timescale for GP1992 was determined by matching the δ^18^O_ice_ profile to the CH_4_ concentrations and δ^18^O_ice_ records from polar ice cores (*14*). To test the timescale, 45 samples were cut throughout the 308-m core and sent to ETH Zurich for measurements of ^36^Cl concentrations.

During a 2015 cooperative field program between the BPCRC and the ITP, five cores were drilled to bedrock on the GIC, two on the GP and three on the GS. Permission to collect the samples was granted by the ITP, Chinese Academy of Sciences as part of the cooperative program under the auspices of the Third Pole Environmental Program. Like the GP1992 ice core, the 309.7-m GP2015 core was split equally between the BPCRC and the ITP, and the BPCRC core was returned frozen to OSU and stored in the cold storage facility at −35°C. GP2015 was cut into 9329 samples for stable isotopes of water which were measured using a Picarro cavity ring-down spectrometer (L2120-i) with an average precision of 0.25‰ for δ^18^O_ice_. Chloride concentrations were measured with a Thermo Fisher Scientific Dionex ion chromatograph (ICS-5000). Timescale development for the GP2015 record to 41 ka was described in (*15*, *16*).

### ^10^Be and ^36^Cl preparation and analysis

To confirm the results of the ^36^Cl data from GP1992, samples from GP2015 were cut throughout the core at BPCRC and sent to the Laboratory for Ion Beam Physics at ETH Zurich, Switzerland, for ^36^Cl and ^10^Be analysis. The sample weights ranged from 0.5 to 0.9 kg of ice. Sample preparation for ^10^Be followed the procedure described in (*57*). The ice samples were passively melted, and 0.1875 mg of ^9^Be carrier was added to each. For ^10^Be measurements, the compact 300-kV AMS system MILEA was used (*58*). The measured ^10^Be/^9^Be ratios were normalized to the ETH in-house standard S2007N with a nominal ratio of ^10^Be/^9^Be = (28.1 ± 0.8) × 10^−12^ (*59*), which in turn was normalized against the ICN 01-5-1 standard with a nominal value of ^10^Be/^9^Be = 2.709 × 10^−11^ (*60*). Typical measurement uncertainties of ^10^Be concentrations were 3 to 8%.

Sample preparation for ^36^Cl followed the procedure described in (*52*). Samples were prepared from the same ice samples as ^10^Be, and 4 mg of natural Cl carrier was added. The AMS measurement of ^36^Cl was performed at the 6 MV Tandem Accelerator using a gas-filled magnet for additional suppression of the isobaric interference of ^36^S (*61*). All ^36^Cl/Cl ratios were normalized with the in-house standard K382/4N, which has a nominal value of ^36^Cl/Cl = (17.36 ± 0.34) × 10^−12^ (*59*), which was normalized to the primary standard KNSTD5000 with a nominal ratio of ^36^Cl/Cl = 5.00 × 10^−12^ (*62*). Typical measurement uncertainties for ^36^Cl were 3 to 15% but were larger for bottom ice with lower ^36^Cl concentrations. All ^10^Be and ^36^Cl measurements were blank corrected with Milli-Q water blanks prepared in parallel to the respective samples.

### ^14^C-WIOC sample collection, preparation, and analysis

Ice samples were collected in the summer of 2015 from the base of a vertical ice cliff at the margin of the GIC (fig. S6). At the sampling location, a 1.5- to 2-m-deep cavity was excavated in the ice ∼50 cm above the base using a chainsaw. The surface layer of the ice wall, potentially affected by melting, was removed. A cubic ice sample with a side length of ∼40 cm and showing distinct horizontal ice layers was obtained from the cave. The ice block was immediately wrapped in clean plastic, transported to the laboratory, and stored at −20°C until further analysis.

The ice block was subsampled from the middle into two samples (∼1 kg each), an upper and a lower one, for radiocarbon analysis. They were prepared following the protocol described in (*28*), with a summary provided here. To remove potential contamination, precut samples from the inner part of the ice block were rinsed with ultrapure water (Sartorius, 18.2 MΩ cm). To remove carbonate potentially present in the ice, melted samples were acidified with HCl to pH < 2 before being sonicated for 5 min. The meltwater was passed through prebaked (heated at 800°C for 4 hours) quartz fiber filters (Pallflex Tissueqtz-2500QAT-UP). In a second carbonate removal step, the filters were acidified three times with a total of 50 μl of 0.2 M HCl, left for 1 hour, rinsed with 5 ml of ultrapure water, and allowed to dry.

Radiocarbon measurements were conducted at the Xi’an Accelerator Mass Spectrometer Center, Institute of Earth Environment, Chinese Academy of Sciences, with a precision of 0.2 to 0.3% and an accuracy of 3 to 4‰, based on long-term standards (*63*, *64*). Filters were then combusted at 600°C and graphitized offline. Graphitized samples were then measured with a 200-kV compact accelerator mass spectrometer (MIni CArbon DAting System, MICADAS). All ^14^C results are expressed as fraction modern (F^14^C), which is the ^14^C/^12^C ratio of the sample divided by the same ratio of the modern standard referenced to the year 1950 (NIST, SRM 4990C, oxalic acid II), both being normalized to −25‰ in δ^13^C to account for isotopic fractionation. All F^14^C values are corrected for blanks. To obtain enough carbon for blank measurements, three filters were combined into one blank sample. For ^14^C-WIOC analysis, a correction is included for constant contamination (10 ± 6 μgC with F^14^C of 0.45 ± 0.11, nine blanks). The online program OxCal v4.4.4 with the IntCal20 calibration curve was used to calibrate the ^14^C ages (*65*, *66*). All calibrated ages, given in years before the present, are indicated with calendar year before the present, and the 1σ range is noted unless stated otherwise.
