# Supplementary material for: Additional evidence supports a timescale exceeding 100,000 years for the Guliya ice cap, Tibetan Plateau
Source: Sci Adv. 2026 Sep 30;12(40):eaeh2912. doi: 10.1126/sciadv.aeh2912 (PMC13626085; doi:10.1126/sciadv.aeh2912)
Supplement: Supplementary file 1 [file sciadv.aeh2912_sm.pdf]

Science Advances
AAAS

Supplementary Materials for

Additional evidence supports a timescale exceeding 100,000 years for the Guliya ice cap, Tibetan Plateau

Lonnie G. Thompson *et al.*

Corresponding author: Lonnie G. Thompson, thompson.3@osu.edu

Sci. Adv. **12**, eaeh2912 (2026)
DOI: 10.1126/sciadv.aeh2912

This PDF file includes:

Figs. S1 to S11

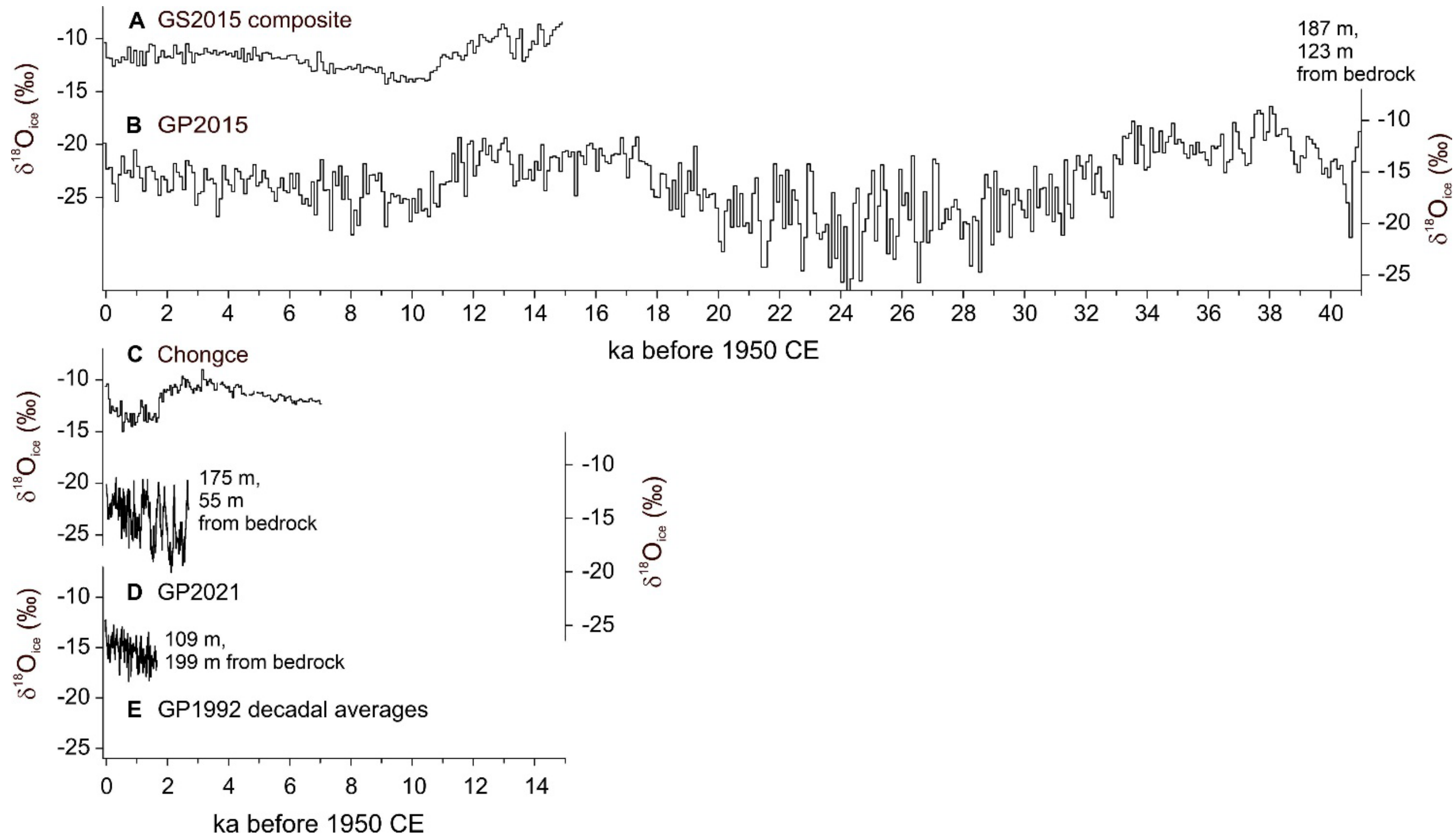

Fig. S1. $\delta^{18}O_{ice}$ records from cores drilled on the western Kunlun ice sheet. (**A**) a composite record of two cores from the summit of the GIC extending back to 15 ka (*15*); (**B**) the 41 kyr record from the plateau of the GIC (*16*); (**C**) a record of $\delta^{18}O_{ice}$ from cores drilled on the Chongce ice cap (*19*); (**D**) a 2.7 kyr $\delta^{18}O_{ice}$ record from the GIC (*22*); (**E**) decadal averages of $\delta^{18}O_{ice}$ in GP1992 back to 1.65 ka (*23*), the timescale for which was determined by mapping visible annual stratigraphy (i.e., dry season dust layers).

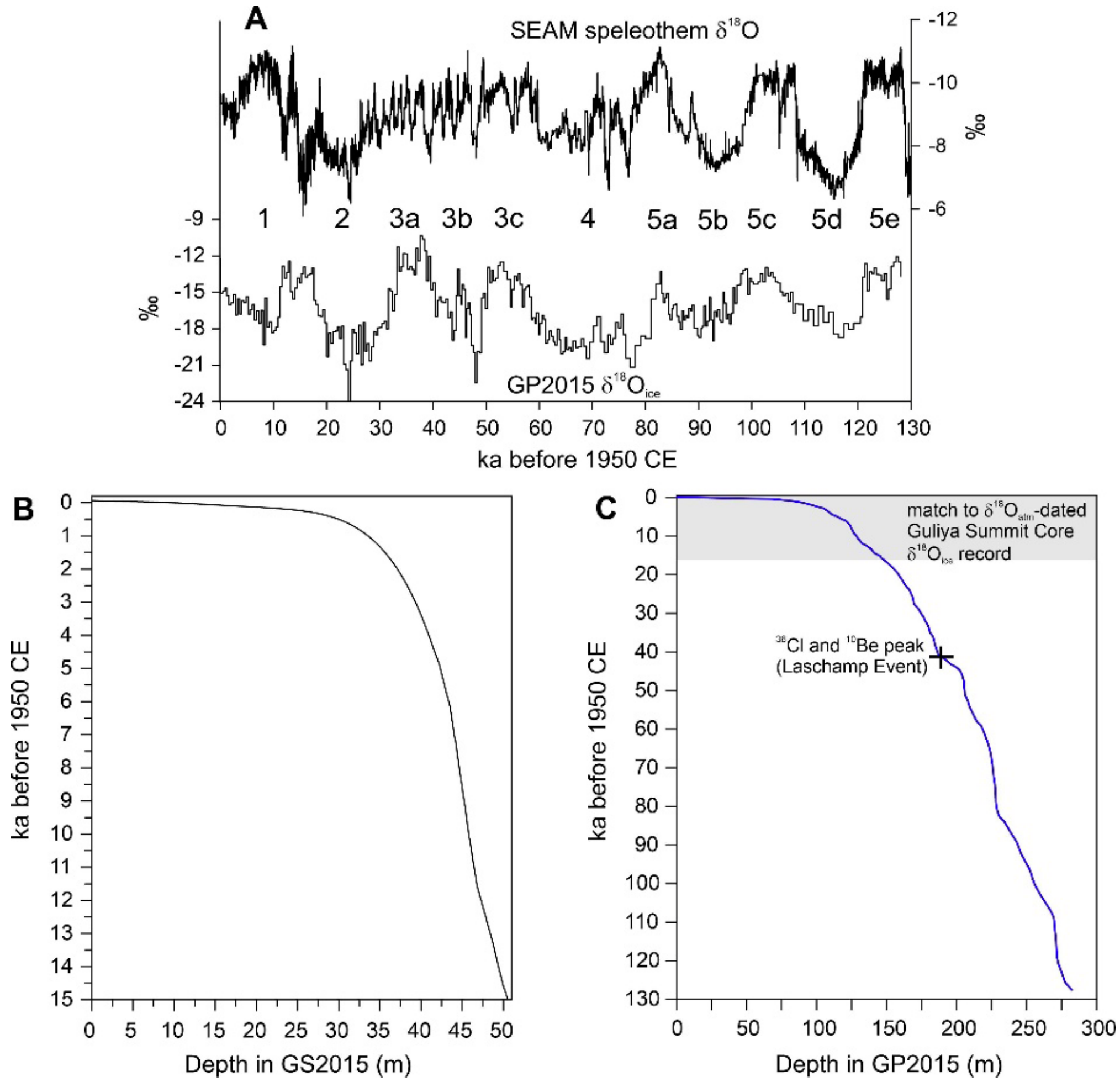

Fig. S2. Extension of the GP1992/2015 timescale to 128 ka. (**A**) The composite GP1992/2025 $\delta^{18}O_{ice}$ record is compared with the composite speleothem record of SE Asian Monsoon activity (*25*). (**B**) Age-depth relationship of the $\delta^{18}O_{atm}$-dated 15-kyr Guliya Summit Core composite. (**C**) Age-depth relationship as a basis for the 128 kyr timescale for GP2015. Grey shading indicates the portion of the core dated by $\delta^{18}O_{ice}$ matching with the $\delta^{18}O_{atm}$ dated Guliya Summit Core record (**B**) (*15*). The age-depth of the cosmogenic isotope peak identified as the Laschamp Event is marked.

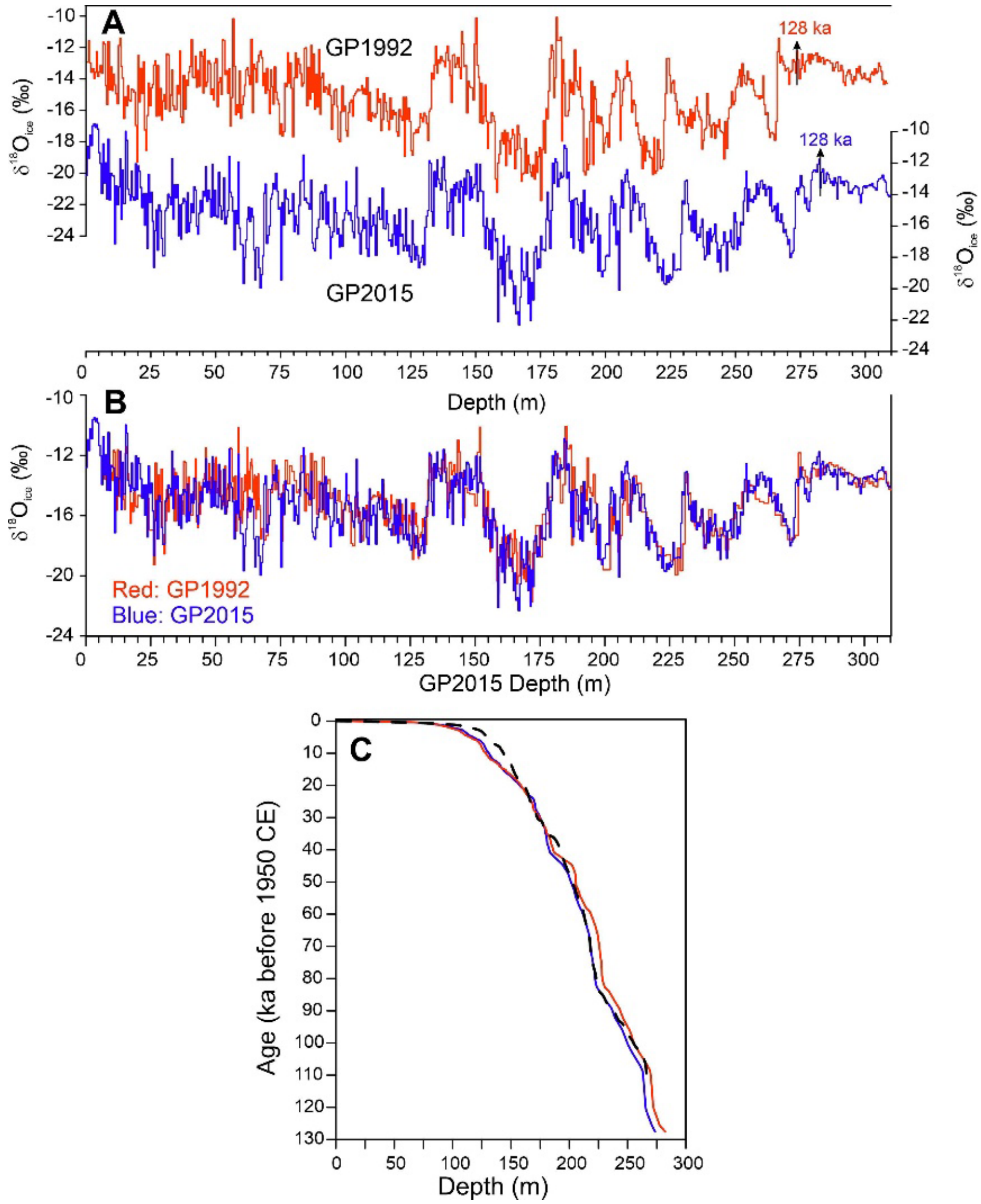

Fig S3. Timescales for GP1992 and GP2015 to 128 ka. (**A**) Comparison between 50-cm averages of $\delta^{18}O_{ice}$ for GP1992 (red) and GP2015 (blue), the depth at which 128 ka occurs is marked in each. (**B**) GP2015 $\delta^{18}O_{ice}$ (50-cm averages) overlain with GP1992 $\delta^{18}O_{ice}$ (50-cm averages) that have been converted to GP2015 depth. (**C**) Depth vs age for GP1992 (red curve) and GP2015 (blue curve). The depth vs age relationship for the original timescale (*14*) is shown as a dashed black curve.

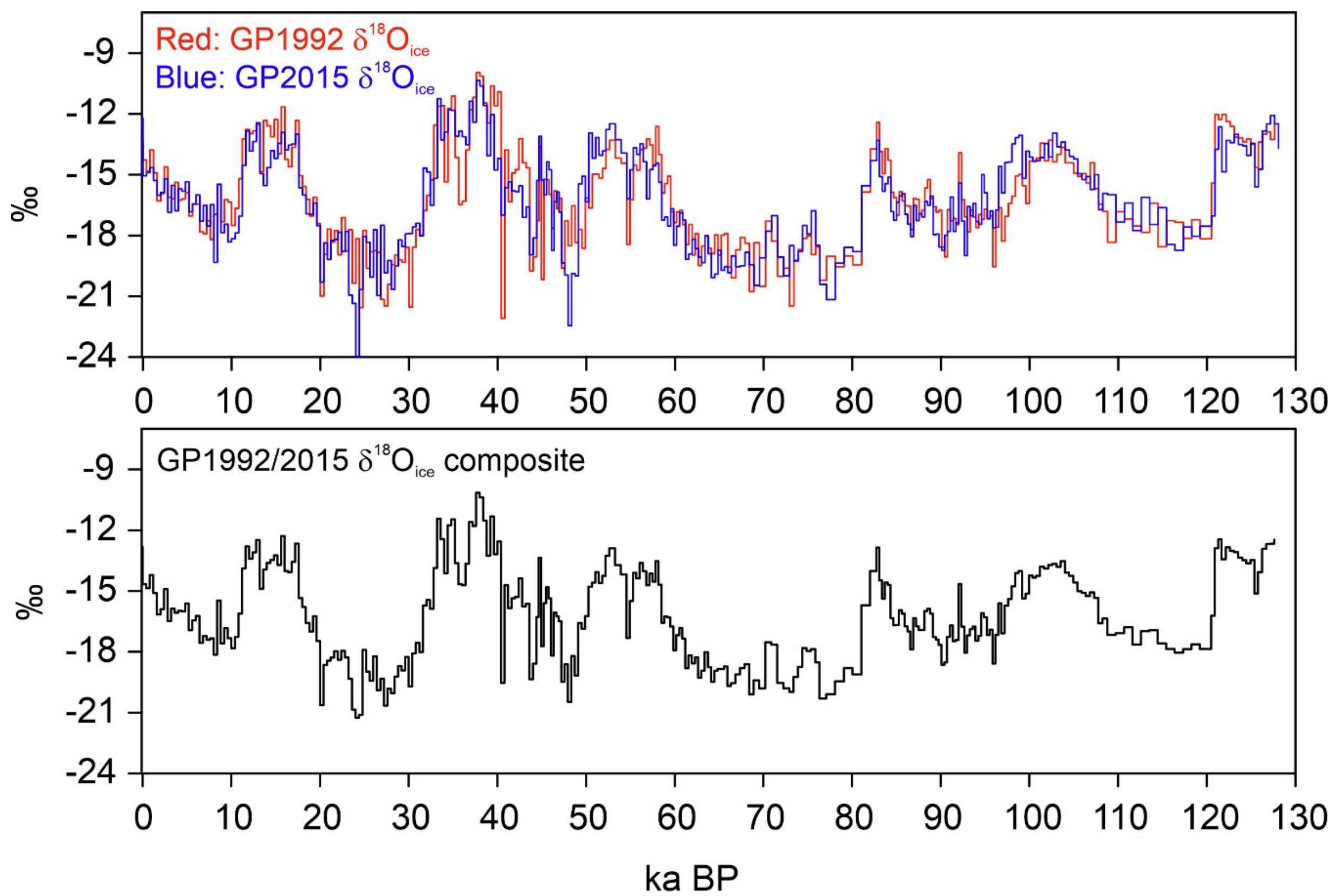

Fig. S4. Comparison of the 128 kyr records of $\delta^{18}O_{ice}$ from GP1992 and GP2015 and the average of the two records to create the GP1992/2015 composite.

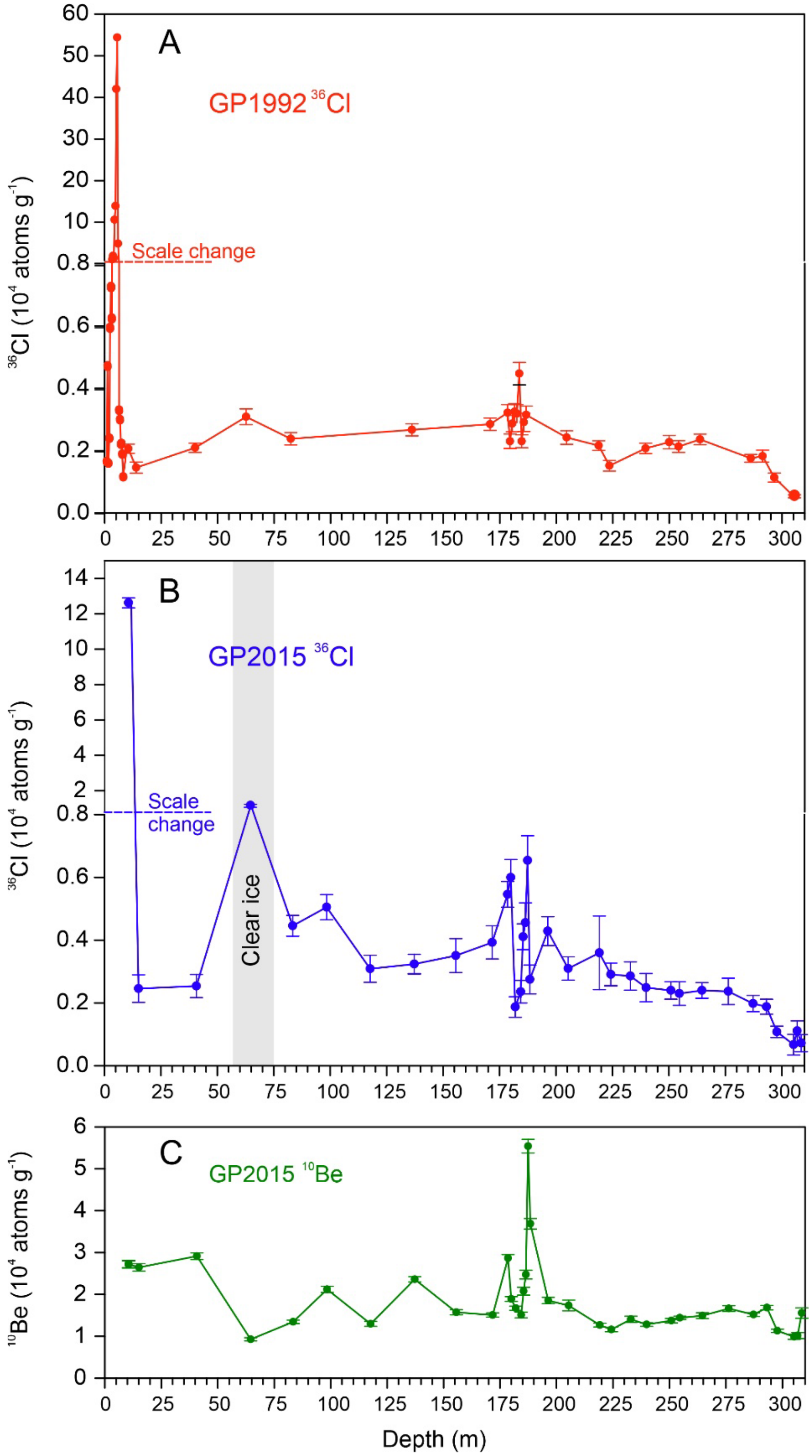

Fig. S5. Cosmogenic isotopes in GP1992 and GP2015. Concentrations of ^{36}Cl from (**A**) GP1992 and (**B**) GP2015, and (**C**) ^{10}Be concentrations from GP2015. Near-surface ^{36}Cl concentrations in GP1992 (*42*) and GP2015 are plotted on different scales than the rest of their respective profiles.

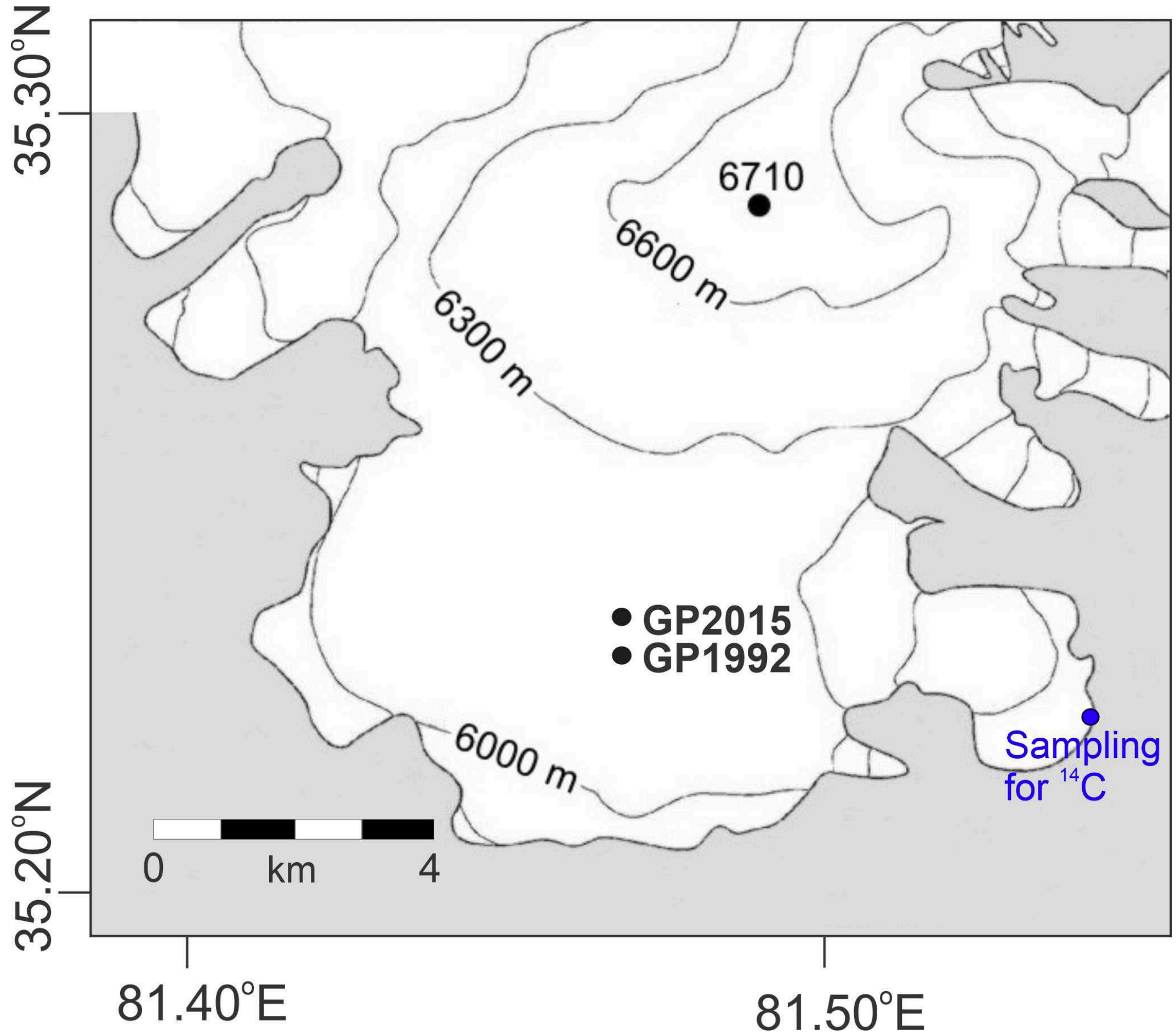

Fig. S6. Location of samples collected in 2015 by ITP for ^{14}C-WIOC measurements. Also shown are the GP1992 and GP2015 drilling locations.

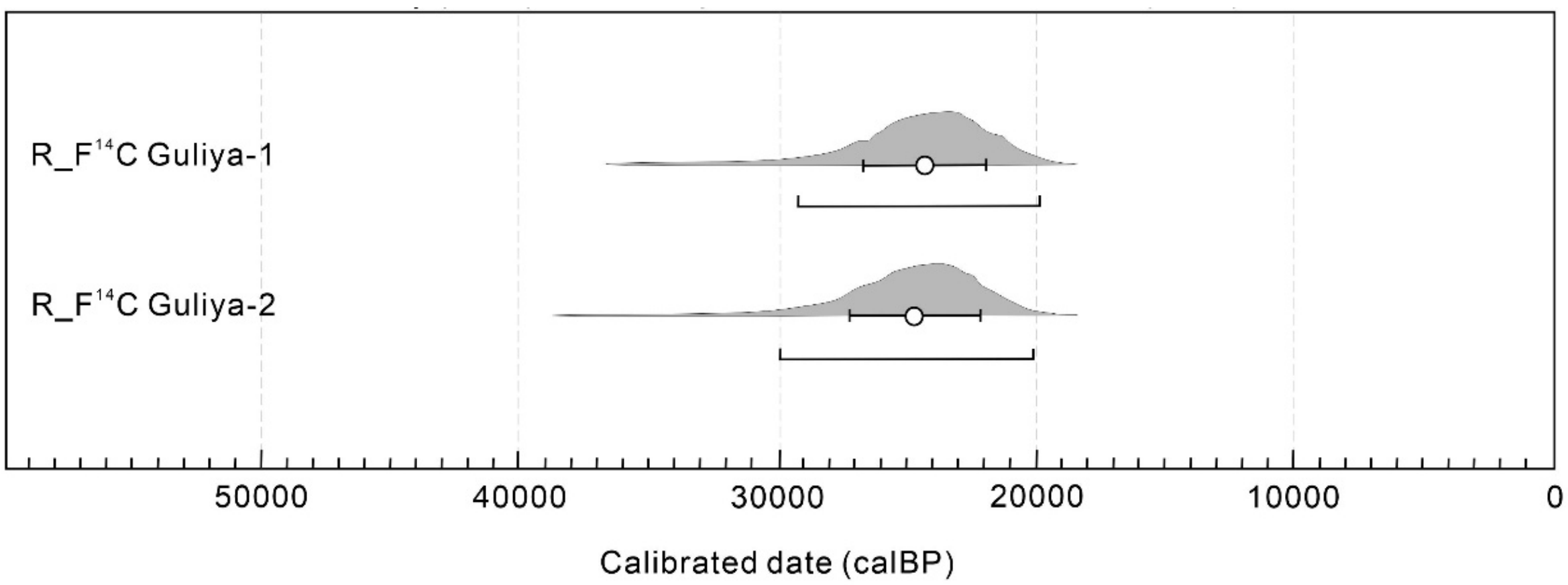

Fig. S7. Radiocarbon ages of samples from the southeastern Guliya margin. The ^{14}C calibration results are based on OxCal v. 4.4.4 (*66*) using the IntCal20 calibration curve (*67*). The possible age distribution is indicated by the gray shaded area. The lines below the histograms indicate the 2σ range of possible age distribution. The mean and 1σ range are shown as open circles and error bars, respectively. Note that reversal of the x-axis (age) in this plot.

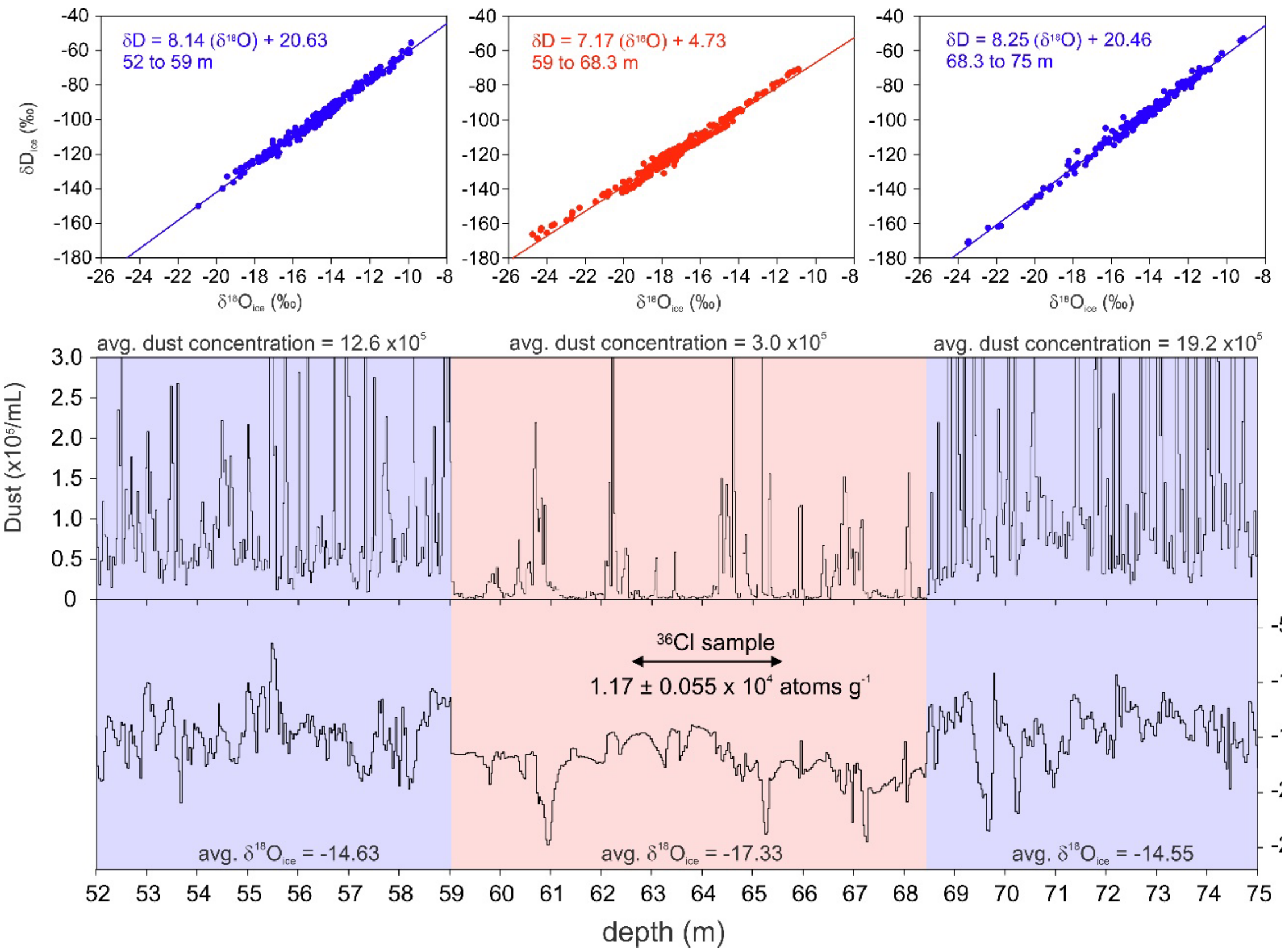

Fig. S8. ^{36}Cl concentration in section of anomalous stratigraphy in GP2015. Dust concentration and $\delta^{18}O_{ice}$ data from 52 to 75 m in GP2015 show decreases in both parameters between 59 and 68.5 m whereas the ^{36}Cl concentration is the highest in the core, with the exception of the ^{36}Cl concentration at 10.5 m (Fig. 2**A**, fig. S5**B**). The plots of meteoric water lines above show that the relationship between $\delta^{18}O_{ice}$ and δD_{ice} is unusual in this section.

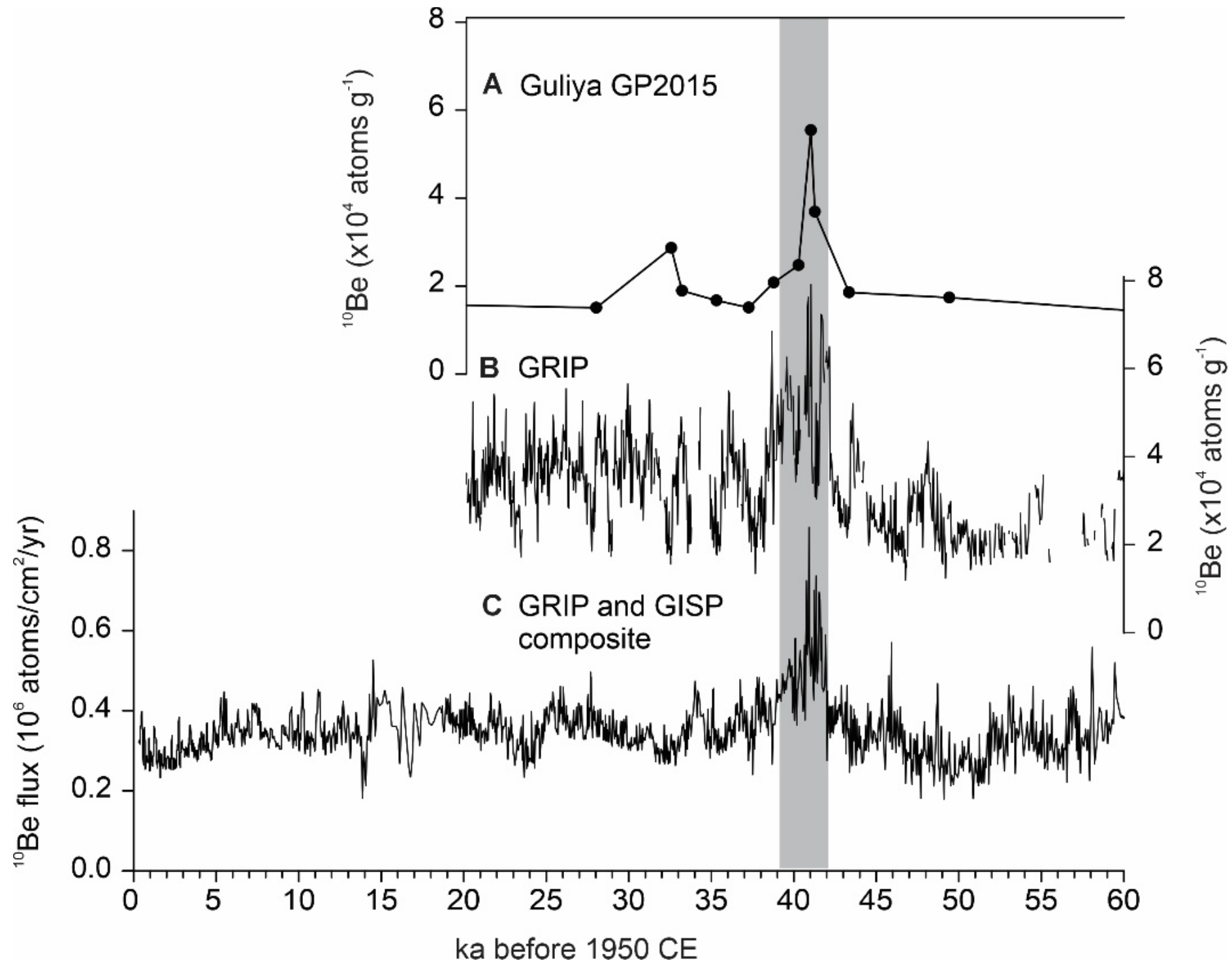

Fig. S9. Comparison between GP2015 ^{10}Be concentrations and Greenland ^{10}Be records (*44*). (**A**) The GP2015 ^{10}Be concentrations; (**B**) ^{10}Be concentrations from the GRIP Greenland ice core; (**C**) composite ^{10}Be flux record from the GRIP and GISP Greenland ice cores. The Laschamp Geomagnetic Excursion (LGE, ~41 ka) is indicated by grey shading.

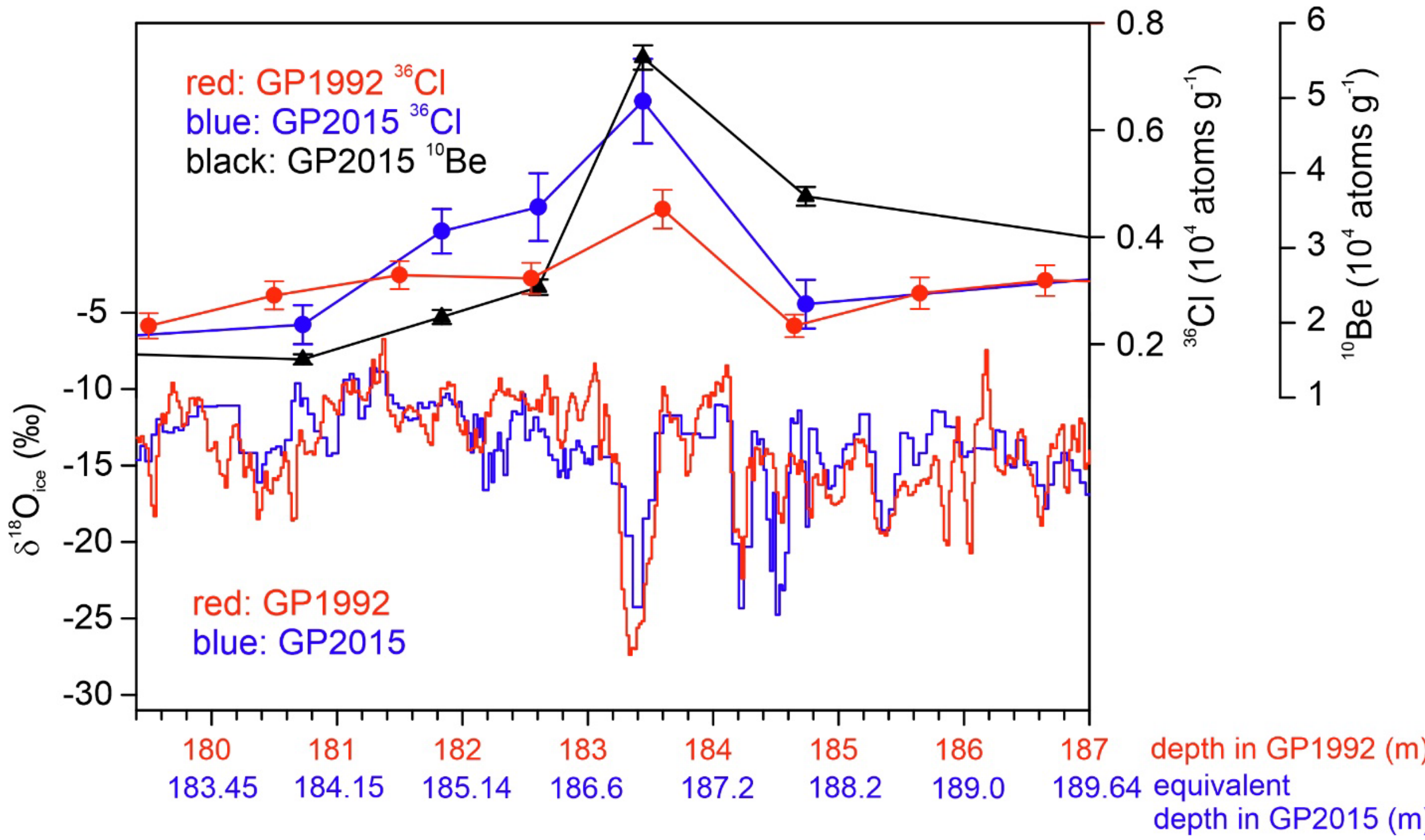

Fig. S10. Climate cooling during the LGE. ^{36}Cl and $\delta^{18}O_{ice}$ data from GP1992 (red plots) and GP2015 (blue plots) and ^{10}Be from GP2015 (black plot) within the respective depth intervals containing the designated LGE (~41 ka).

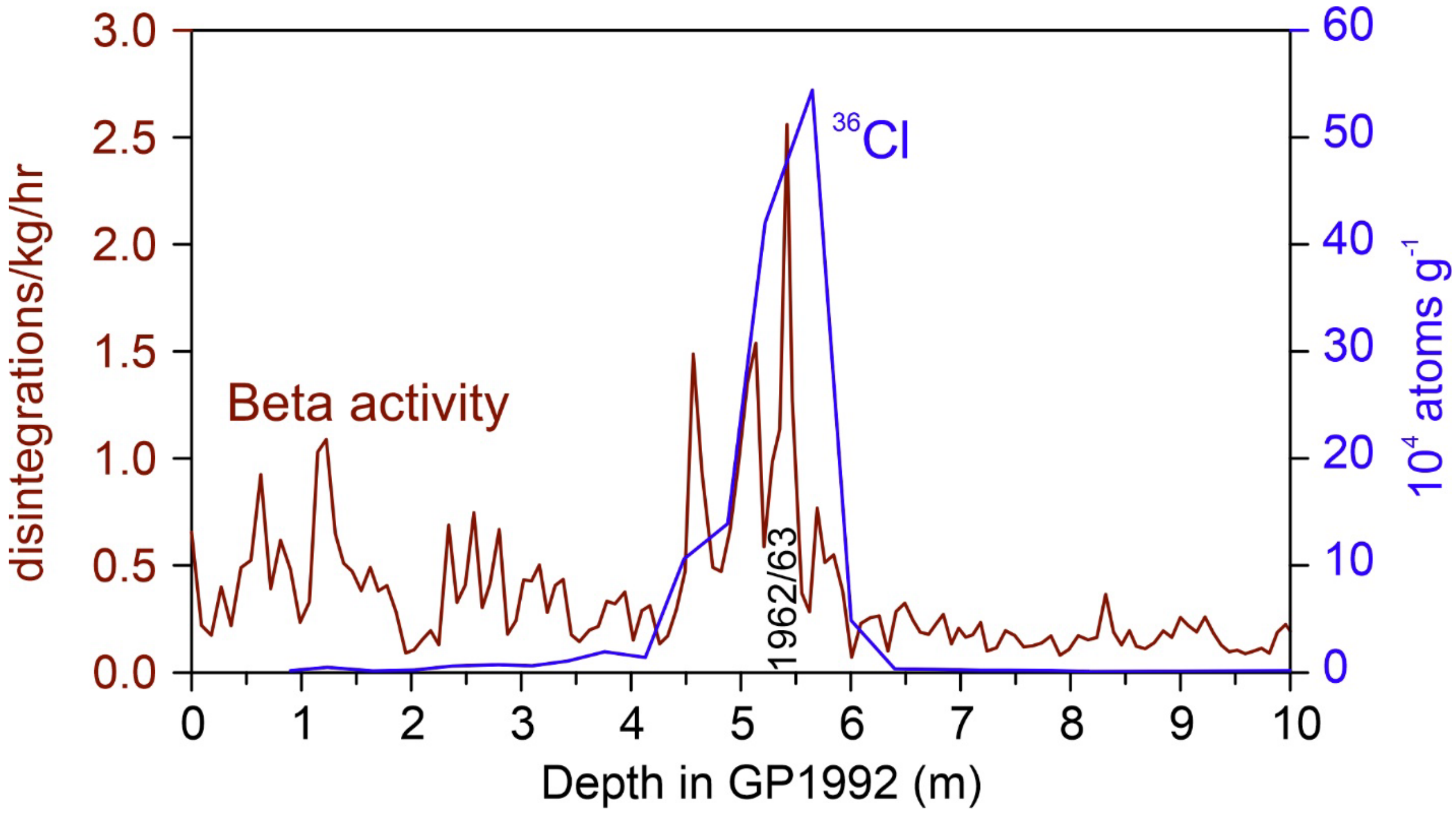

Fig. S11. ^{36}Cl and Beta emissions fallout in the GP1992 core (*42*). Both peaks result from late 1950s to 1960s atmospheric thermonuclear tests.
